# Spinal cord compression by B-cell lymphoma, unclassifiable, with features intermediate between diffuse large B-cell lymphoma and Burkitt lymphoma in a patient seropositive for human immunodeficiency virus: a case report

**DOI:** 10.1186/1752-1947-8-324

**Published:** 2014-10-01

**Authors:** Jun-Yeong Seo, Kee-Yong Ha, Min-Up Kim, Yoon-Chung Kim, Young-Hoon Kim

**Affiliations:** 1 Department of Orthopaedic Surgery, Jeju National University Hospital, Aran 13gil 15, Jeju 690-767, South Korea; 2 Department of Orthopaedic Surgery, Seoul St. Mary’s Hospital, College of Medicine, The Catholic University of Korea, 222 Banpo-Daero, Seocho-Gu 137-701 Seoul, South Korea

**Keywords:** Acquired immune deficiency syndrome, B cell lymphoma, Burkitt, Non-Hodgkin lymphoma, Spinal cord compression

## Abstract

**Introduction:**

Although non-Hodgkin’s lymphoma is one of the most common and frequently fatal of the acquired immune deficiency syndrome-defining illnesses, survival has improved significantly since the introduction of antiretroviral therapy. Patients with spinal cord compression resulting from non-Hodgkin’s lymphoma present with clinically acute or rapidly progressive neurologic deficits. The purpose of this case report is to present a case of a patient seropositive for human immunodeficiency virus with spinal cord compression due to B-cell lymphoma, unclassifiable, with features intermediate between diffuse large B-cell lymphoma and Burkitt lymphoma.

**Case presentation:**

A 40-year-old Asian man, who was seropositive for human immunodeficiency virus, presented with progressive neurological deficits. Magnetic resonance images of his thoracic spine showed an epidural mass from T2 to T4, resulting in severe cord compression. Emergent surgical decompression and biopsy were performed, followed by palliative radiation therapy. The pathologic findings showed that the specimen was compatible with B-cell lymphoma, unclassifiable, with features intermediate between diffuse large B-cell lymphoma and Burkitt lymphoma. Palliative radiation therapy was performed; however, leptomeningeal seeding and pulmonary embolism led to his death.

**Conclusions:**

When a patient infected with human immunodeficiency virus presents with a rapidly progressive spinal tumor accompanying paraplegia, non-Hodgkin’s lymphoma should be considered, and surgical decompression should be weighed with respect to the patient’s general condition and the subtype/prognosis of the lymphoma.

## Introduction

The 2008 World Health Organization classification system of tumors of hematopoietic and lymphoid tissue included an overlap category termed B-cell lymphoma, unclassifiable (B-UCL), with features intermediate between diffuse large B-cell lymphoma (DLBCL) and Burkitt lymphoma (BL) [1]. Previously classified as Burkitt-like lymphomas, this nonhomogeneous category encompasses several types of aggressive B-cell lymphoma that are often difficult to diagnose due to the lack of specific morphologic, genetic and immunophenotypic patterns. They do not respond to BL- or DLBCL-type chemotherapeutic regimens, and no treatment consensus in patients with B-UCL has been determined [2]. We present a case of a patient with spinal cord compression caused by an acquired immune deficiency syndrome (AIDS)-related B-UCL with features intermediate between DLBCL and BL.

## Case presentation

**Figure 1:**
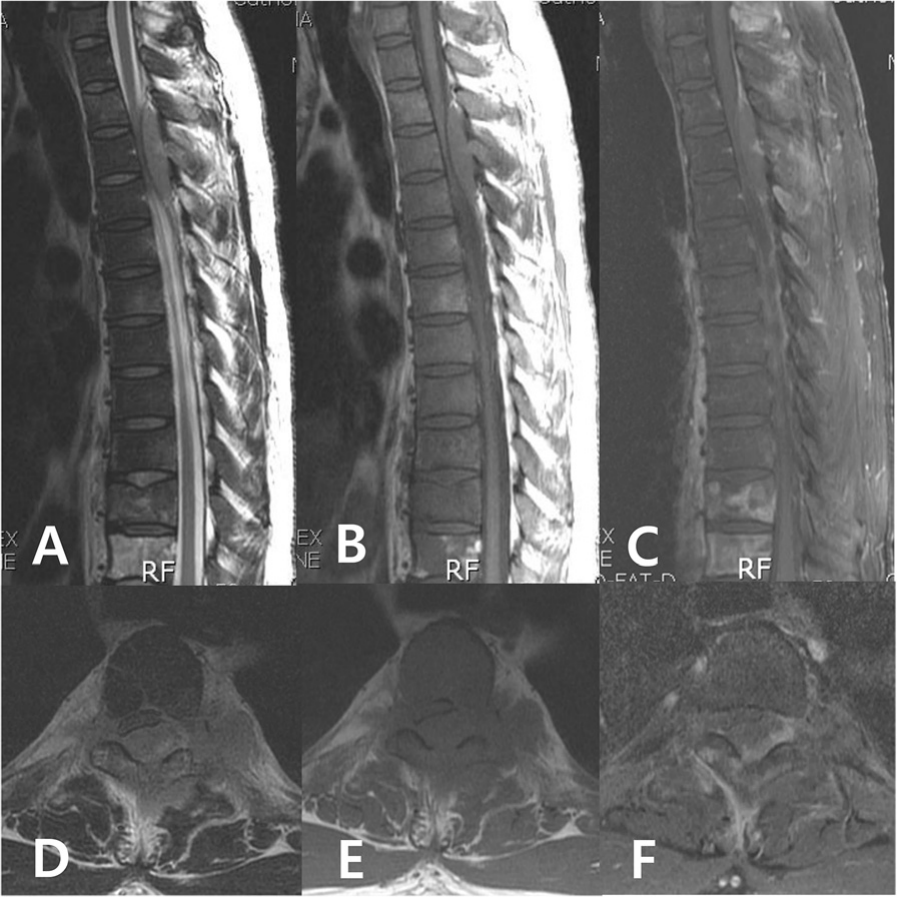
**Magnetic resonance image shows elongated epidural mass at the left posterolateral aspect of the spinal cord at the T2 to T4 levels, resulting in severe cord compression. A**, T2-weighted image sagittal; **B**, T1-weighted image sagittal; **C**, T1-weighted image enhanced sagittal; **D**, T2-weighted image axial; **E**, T1-weighted image axial; **F**, T1-wighted image enhanced axial.

**Figure 2:**
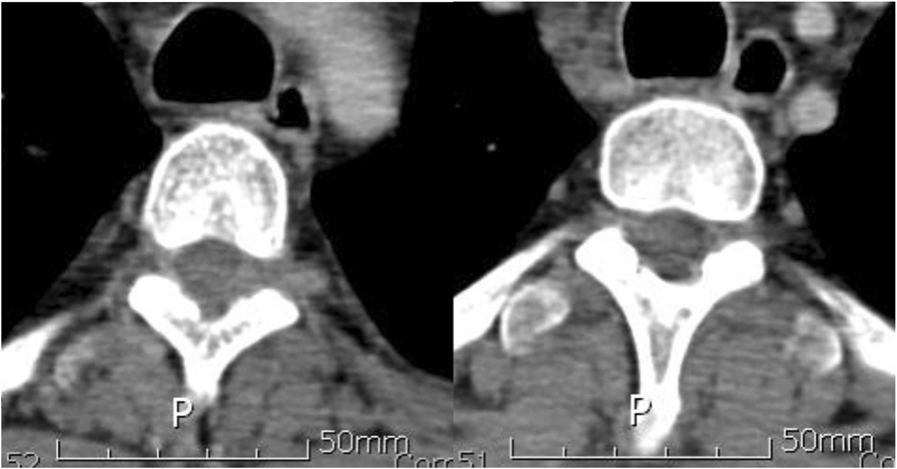
Computed tomography shows no bony destruction at the T2 to T4 levels.

**Figure 3:**
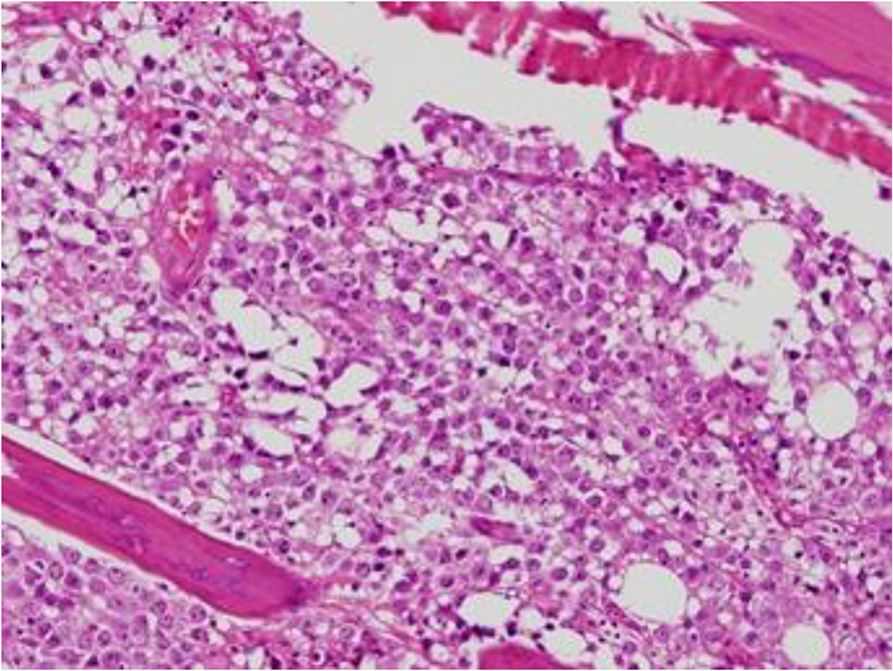
**The pathology findings showed that the specimen was compatible with B-cell lymphoma unclassifiable with features intermediate between diffuse large B-cell lymphoma and Burkitt lymphoma.** Hypercellular bone marrow was replaced by a homogeneous population of lymphoid cells (hematoxylin-eosin stain ×400).

A 40-year-old Asian man complained of progressive pain and weakness in his lower extremities. A physical examination showed decreased muscle power (Frankel grade D), increased sensory loss below the T6 dermatome, ankle clonus and abnormal Babinski reflex. He was diagnosed as being seropositive for human immunodeficiency virus (HIV) 6 months ago, and having AIDS-related lymphoma (ARL) in his liver and an intrahepatic bile duct obstruction 2 months ago. A liver biopsy showed B-cell type lymphoma. He received the chemotherapy combination of cyclophosphamide, doxorubicin, vincristine, and prednisone (CHOP). Magnetic resonance imaging (MRI) of his thoracic and lumbar spine showed a 1.5×2×5cm elongated intraspinal extramedullary mass from T2 to T4. The lesion showed intermediate-to-high signal intensity on T2-weighted image, intermediate-to-low signal intensity on T1-weighted image and heterogeneous enhancement after gadolinium-infusion (Figure 1). A computed tomography (CT) scan showed no definite bony destruction (Figure 2), but abnormal signal intensities and enhancement were found from the T9 to T11 vertebral bodies. Lymphoma was suspected. Emergent radiation therapy was performed at the C7 to T5 field, chemotherapy was administered preoperatively, and surgical decompression and excisional biopsy were performed. The pathologic findings showed that the specimen was compatible with B-UCL with features intermediate between DLBCL and BL (Figure 3). Immunohistochemistry showed the tumor cells were CD20-positive and CD45RO-negative. The patient received chemotherapy and radiation therapy with highly active antiretroviral therapy (HAART) after surgery. Radiation treatment of 200cGy per fraction was performed (3000cGy in 15 fractions). However, his motor power was not improved. A postoperative abdominal CT scan at 5 weeks showed increased lymphoma size in his liver. Moreover, pulmonary thromboembolism and leptomeningeal seeding were detected. A relapsed mass was found from the C5 to T1 area on follow-up MRI. His complete blood cell count was below the lower limit, and his deteriorating condition did not permit additional chemotherapy. He was transferred to hospice care, and he died by massive pulmonary thromboembolism at 13 weeks postoperatively.

## Discussion

HIV infection can result in neoplasm due to damaged cellular immunity [3,4]. Individuals infected with HIV developed lymphoma at a greater rate than the population. Approximately two-thirds of ARL cases are categorized as diffuse large B-cell type, with BL comprising 25% and other histology a much smaller proportion [5]. Recent clinical trials have demonstrated a better outcome with chemotherapy for ARL since the introduction of combination antiretroviral treatment, termed HAART [6]. However, the treatment of lymphomas in patients infected with HIV has been less than satisfactory, with a high mortality rate [4]. The patient in this case was diagnosed with B-UCL with features intermediate between DLBCL and BL. These rare lymphomas, which occur predominantly in adults, have a germinal center phenotype that resembles BL but exhibit atypical cytological features for BL [7]. On morphological examination, cases of B-UCL have architectural and cytological features intermediate between DLBCL and BL, and are composed of a diffuse proliferation of predominantly medium-sized, centroblast-like cells with numerous tingible-body macrophages admixed. Immunophenotypically, these lymphomas often have a germinal center B-cell phenotype with positivity for CD10 and bcl6, and are frequently bcl2 positive. A case of typical BL with strong bcl2 expression is also placed into this category [1]. Clinically, B-UCL is generally considered to be an aggressive lymphoma and most patients present with widespread disease. However, there are currently no well-established therapies for these patients. Most studies have reported poor outcomes with the standard therapies used for DLBCL, as well as with more intensive regimens [1,8-11].

Extranodal involvement of lymphoma commonly involves the gastrointestinal tract, bone marrow, liver, lung and central nervous system. Although survival from ARL has improved significantly with HAART [12], it is associated with high mortality rates when the epidural space is involved [13]. Epidural spinal cord compression occurs in 0.1 to 6.5% of patients with non-Hodgkin’s lymphoma (NHL) [14-16], either at the time of recurrence [17] or at the initial diagnosis [18]. Lymphoma is thought to involve the paraspinal soft tissues first, such as the paravertebral ganglion or epidural lymphoid tissue, and then invade around the cord via the vertebral foramen without destroying bony structures [15,19]. It is important to discriminate whether the paraspinal mass is a primary lesion or secondary metastatic lesion. Molecular imaging by positron emission tomography is helpful to diagnosis, identification of the metabolically active tumor compartment, and prediction of treatment response [20]. Surgery, radiotherapy, or a combination of both are treatment options for spinal cord compression by lymphoma. Chang *et al.*[21] suggested that surgical decompression can improve recovery from neurological deficits in patients with DLBCL-associated spinal cord compression, but Peng *et al.*[22] recommended nonsurgical rather than surgical treatment because of the high mortality rate after surgery. In the present case, the lymphoma metastasized to the epidural space followed by spinal cord compression. The patient did not respond to CHOP chemotherapy and radiation therapy. We were obliged to perform surgical decompression in this urgent situation.

## Conclusions

Spine surgeons should consider the possibility of NHL-associated spinal cord compression in patients seropositive for HIV who present with rapidly progressive paraplegia, and treatment should take into account the patient’s condition in addition to the subtype and prognosis of the lymphoma.

## Consent

Written informed consent was obtained from the patient’s next-of-kin for publication of this case report and any accompanying images. A copy of the written consent is available for review by the Editor-in-Chief of this journal.

## Abbreviations

AIDS: Acquired immune deficiency syndrome; ARL: AIDS-related lymphoma; BL: Burkitt lymphoma; B-UCL: B-cell lymphoma, unclassifiable; CHOP: Chemotherapy combination of cyclophosphamide, doxorubicin, vincristine, prednisone; CT: Computed tomography; DLBCL: Diffuse large B-cell lymphoma; HAART: Highly active antiretroviral therapy; HIV: Human immunodeficiency virus; MRI: Magnetic resonance imaging; NHL: Non-Hodgkin’s lymphoma.

## Competing interests

The authors declare that they have no competing interests.

## Authors’ contributions

MUK and YCK analyzed and interpreted the patient data. JYS was a major contributor in writing the manuscript. KYH and YHK supervised the manuscript. All authors read and approved the final manuscript.
